# Early Effects of Abaloparatide on Bone Formation and Resorption Indices in Postmenopausal Women With Osteoporosis

**DOI:** 10.1002/jbmr.4243

**Published:** 2021-01-28

**Authors:** David W Dempster, Hua Zhou, Sudhaker D Rao, Chris Recknor, Paul D Miller, Benjamin Z Leder, Miriam Annett, Michael S Ominsky, Bruce H Mitlak

**Affiliations:** ^1^ Regional Bone Center Helen Hayes Hospital West Haverstraw NY USA; ^2^ Bone & Mineral Research Laboratory Henry Ford Health System Detroit MI USA; ^3^ United Osteoporosis Centers Gainesville GA USA; ^4^ Colorado Center for Bone Health Lakewood CO USA; ^5^ Mass General Hospital Harvard Medical School Boston MA USA; ^6^ Radius Health, Inc. Boston MA USA

**Keywords:** ANABOLICS, BONE HISTOMORPHOMETRY, BONE MODELING AND REMODELING, CLINICAL TRIAL, OSTEOPOROSIS

## Abstract

Anabolic osteoporosis drugs improve bone mineral density by increasing bone formation. The objective of this study was to evaluate the early effects of abaloparatide on indices of bone formation and to assess the effect of abaloparatide on modeling‐based formation (MBF), remodeling‐based formation (RBF), and overflow MBF (oMBF) in transiliac bone biopsies. In this open‐label, single‐arm study, 23 postmenopausal women with osteoporosis were treated with 80 μg abaloparatide daily. Subjects received double fluorochrome labels before treatment and before biopsy collection at 3 months. Change in dynamic histomorphometry indices in four bone envelopes were assessed. Median mineralizing surface per unit of bone surface (MS/BS) increased to 24.7%, 48.7%, 21.4%, and 16.3% of total surface after 3 months of abaloparatide treatment, representing 5.5‐, 5.2‐, 2.8‐, and 12.9‐fold changes, on cancellous, endocortical, intracortical, and periosteal surfaces (*p* < .001 versus baseline for all). Mineral apposition rate (MAR) was significantly increased only on intracortical surfaces. Bone formation rate (BFR/BS) was significantly increased on all four bone envelopes. Significant increases versus baseline were observed in MBF on cancellous, endocortical, and periosteal surfaces, for oMBF on cancellous and endocortical surfaces, and for RBF on cancellous, endocortical, and intracortical surfaces. Overall, modeling‐based formation (MBF + oMBF) accounted for 37% and 23% of the increase in bone‐forming surface on the endocortical and cancellous surfaces, respectively. Changes from baseline in serum biomarkers of bone turnover at either month 1 or month 3 were generally good surrogates for changes in histomorphometric endpoints. In conclusion, treatment with abaloparatide for 3 months stimulated bone formation on cancellous, endocortical, intracortical, and periosteal envelopes in transiliac bone biopsies obtained from postmenopausal women with osteoporosis. These increases reflected stimulation of both remodeling‐ and modeling‐based bone formation, further elucidating the mechanisms by which abaloparatide improves bone mass and lowers fracture risk. © 2021 The Authors. Journal of Bone and Mineral Research published by Wiley Periodicals LLC on behalf of American Society for Bone and Mineral Research (ASBMR).

## Introduction

Osteoporosis is a disease of the skeleton characterized by deterioration of the microarchitectural structure of bone tissue and loss of bone mass, leading to an increased risk of fracture.^(^ ^1^ ^)^ Throughout life, bone tissue is continuously renewed through cycles of bone remodeling, which involves resorption of bone by osteoclasts, followed by formation of new bone over resorbed surfaces, a process that becomes imbalanced during aging.^(^ ^2^ ^)^ In contrast, the actions of osteoblasts are not coupled to osteoclasts during modeling‐based bone formation, which is responsible for the formation of new bone that occurs during initial skeletal development and in response to loading and other stimuli in adults.^(^ ^3^, ^4^ ^)^

Most osteoporosis drugs are antiresorptive agents (bisphosphonates, denosumab, selective estrogen receptor modulators [SERMs], calcitonin, and estrogens), which improve bone mineral density (BMD) through suppression of osteoclast‐mediated bone resorption during bone remodeling.^(^ ^5^, ^6^ ^)^ Conversely, anabolic agents, including abaloparatide, teriparatide, and romosozumab, increase BMD by shifting the balance during bone remodeling to favor osteoblast‐mediated bone formation and by stimulating modeling‐based formation (MBF), that is, formation without preceding resorption.^(^ ^3^, ^4^, ^6^, ^7^, ^8^ ^)^

Abaloparatide is an anabolic agent that increased BMD and reduced the risk of vertebral and nonvertebral fractures in postmenopausal women with osteoporosis in the pivotal Abaloparatide Comparator Trial in Vertebral Endpoints (ACTIVE) trial.^(^ ^9^ ^)^ Biopsies taken from a subset of subjects from ACTIVE (*n* = 105; 78 biopsies analyzed) between 12 and 18 months showed normal bone microarchitecture and no adverse effects on bone quality;^(10^ ^)^ however, bone biopsies were primarily obtained to assess safety and did not provide information on the anabolic effects of abaloparatide.

Quadruple fluorochrome labeling is a technique that has been found to be highly effective in demonstrating early effects of anabolic therapies on bone formation.^(^ ^4^, ^7^, ^11^, ^12^, ^13^ ^)^ The principal advantage of the technique is that it allows measurement of dynamic bone formation variables before and after initiation of a drug treatment in a single biopsy. Using this technique, the short‐term anabolic effects of teriparatide were demonstrated in histomorphometric analyses of single transiliac bone biopsies, including increases in both modeling‐ and remodeling‐based bone formation.^(^ ^4^, ^7^, ^12^ ^)^ Likewise, quadruple fluorochrome‐labeled biopsies taken after 2 months of treatment with romosozumab demonstrated increased modeling‐based bone formation, while biopsies taken at 12 months with conventional double fluorochrome labeling showed reduced bone formation with romosozumab compared with placebo treatment.^(^ ^11^ ^)^ Using a protocol that was very similar to the present study, the Anabolic Versus Antiresorptive (AVA) trial showed that a 3‐month treatment with teriparatide stimulated remodeling‐, modeling‐, and overflow modeling‐based bone formation on the cancellous and endocortical envelopes and also stimulated modeling‐based bone formation on the periosteal surface.^(^ ^4^ ^)^ These tissue‐level changes have been shown to correlate with changes in serum markers of bone formation.^(^ ^14^ ^)^

The objectives of this study were to assess the early effects of abaloparatide in postmenopausal women with osteoporosis using tissue‐based indices of bone remodeling (or turnover) obtained by transiliac bone biopsy after quadruple tetracycline labeling and to relate these indices to biochemical markers of bone turnover.

## Materials and Methods

### Study design

**Fig 1:**
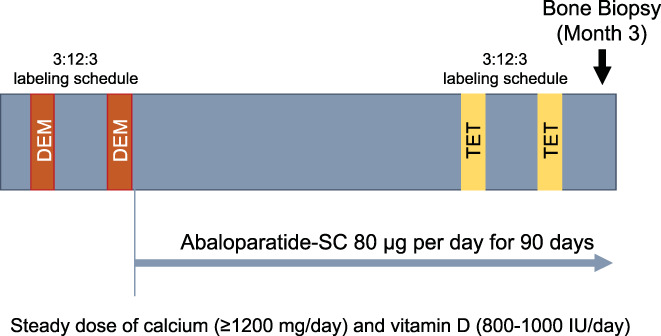
Study design. DEM = demeclocycline; SC = subcutaneous; TET = tetracycline.

This was an open‐label, single‐arm study of postmenopausal women with osteoporosis treated with 80 μg abaloparatide for 3 months. Transiliac bone biopsies were taken at 3 months after quadruple fluorochrome labeling (Fig. 1). The treatment duration of 3 months was determined to be the optimal time when biochemical markers of bone turnover peak and are predictive of subsequent changes in BMD.^(^ ^15^ ^)^ In addition, time points beyond 3 months increase the risk that the initial set of fluorochrome labels would be resorbed by newly activated remodeling units.^(^ ^12^ ^)^

The study was approved by the ethics committee at every participating institution and was conducted according to the recommendations of Good Clinical Practice and the Declaration of Helsinki. All subjects provided written informed consent to participate in the study.

### Study population

Ambulatory postmenopausal (≥5 years) women 50 to 85 years of age (inclusive) with osteoporosis were included if they had a BMD *T*‐score ≤ −2.5 at the lumbar spine or hip (femoral neck or total hip). Subjects with a BMD *T*‐score ≤ −2.0 at the lumbar spine or hip with a history of low‐trauma vertebral, forearm, humerus, sacral, pelvic, hip, femoral, or tibial fracture within 5 years before enrollment were also eligible. All subjects were required to have serum calcium, parathyroid hormone (PTH), phosphorus, and alkaline phosphatase levels within the normal range and serum 25‐hydroxy vitamin D values ≥20 ng/mL and within the normal range.

Subjects were excluded if they had unevaluable lumbar spine or hip BMD; a history of bone disorders (eg, Paget's disease) other than postmenopausal osteoporosis; Cushing's disease, hypo‐ or hyperparathyroidism, or malabsorptive syndromes within the past year; cancer within the past 5 years (other than basal cell or squamous cancer of the skin); osteosarcoma at any time; or prior radiotherapy, other than radioiodine. Subjects with known hypersensitivity to abaloparatide; prior treatment with PTH or PTHrP drugs, denosumab, or IV bisphosphonates; treatment with oral bisphosphonates within the past 3 years; or treatment with calcitonin, SERMs, or tibolone in the past 6 months were not eligible for the study.

### Bone biopsy

Quadruple labeling was performed with the fluorochromes demeclocycline and oxytetracycline as previously described.^(^ ^4^, ^7^, ^12^ ^)^ Briefly, 18 days before abaloparatide treatment, subjects took 150 mg demeclocycline 4 times daily for 3 days followed by a 12‐day intermission, and then demeclocycline for 3 additional days at the same dose. A second set of double labels using 250 mg oxytetracycline was administered 23 to 26 days before bone biopsy collection at month 3, following the same schedule. Transiliac bone biopsies were performed 5 to 8 days after the last oxytetracycline administration using a Rochester or similar large‐bore (6 to 8 mm) manual trephine. Samples were processed without decalcification and embedded in polymethyl methacrylate.^(^ ^4^, ^12^, ^16^ ^)^

Each biopsy section was subjected to histomorphometric measurements using computerized image analysis as previously described.^(^ ^4^, ^17^ ^)^ All variables were calculated and expressed according to the guidelines of the ASBMR's Bone Histomorphometry Committee.^(^ ^18^ ^)^ Three levels of sections from each biopsy block were cut 100 μm apart. In each level, adjacent sections at 7‐ and 20‐μm thickness were collected. The 7‐μm section was stained with toluidine‐blue to visualize osteons and the underlying cement lines. The 20‐μm section was left unstained for visualization of tetracycline labels. All biopsies were analyzed by a single reader (HZ). Bone formation was designated as MBF if the underlying cement line was smooth and remodeling‐based formation (RBF) if the cement line was scalloped. Formation over smooth cement lines adjacent to scalloped reversal lines was considered to be overflow MBF (oMBF). The referent for all indices was bone surface (BS). If only single labels were present, the mineral apposition rate (MAR) was either treated as a missing value or given an imputed value of 0.3 μm/d.^(^ ^18^ ^)^

### Biochemical markers of bone turnover

Blood was collected on day 1, month 1, and month 3 to measure the efficacy‐related markers of bone remodeling or turnover: serum procollagen type I N‐terminal propeptide (s‐PINP) and serum carboxy‐terminal cross‐linking telopeptide of type I collagen (s‐CTX). Fasting was not required before blood collection.

### Safety

Safety assessments included adverse events (AEs), serious adverse events (SAEs), physical examinations, vital signs (blood pressure, body temperature, pulse rate, and respiratory rate), and electrocardiograms (ECGs).

Clinical laboratory tests included hematology, serum chemistry, coagulation, and urinalysis.

### Sample size

In the ACTIVE study,^(^ ^9^ ^)^ the median percentage change from baseline in s‐PINP at 3 months was 60% for abaloparatide and 94% for teriparatide. The ratio between the two groups is approximately two‐thirds. Using this ratio and the results for teriparatide obtained from the AVA study,^(^ ^12^ ^)^ it was assumed that the mean change from baseline in cancellous mineralizing surface per unit of bone surface (MS/BS) (%) for abaloparatide at 3 months would be approximately 9.2%. Assuming a standard deviation (SD) of 12.0%, a sample size of 21 completers would provide at least 90% power to detect a statistically significant change from baseline in cancellous MS/BS (%) for abaloparatide at 3 months of 9.2%. The study planned to enroll approximately 25 subjects to accommodate a drop‐out rate of 15%, including those with unevaluable bone biopsy samples.

### Statistical analyses

The Bone‐Biopsy Population, which included all enrolled subjects who had a bone biopsy, was the primary population for bone histomorphometry and serum biomarker analyses. The primary population for all safety analyses was the Safety Population, which included all enrolled subjects who received at least 1 dose of abaloparatide.

The primary endpoint was the change from baseline to 3 months in MS/BS in the cancellous bone envelope. Paired *t* test was used to compare the differences in dynamic indices between the two time points derived from the two sets of double labels. If the normality assumption for the efficacy data was not satisfied at the 0.01 significance level with Shapiro–Wilk test and if visual inspection of the data deemed it necessary, the Wilcoxon signed‐rank test was used to assess changes from baseline. No adjustments for multiplicity were made. A two‐sided *p* value <.05 was considered statistically significant. Actual values and change from baseline values were summarized with descriptive statistics by visit.

Secondary efficacy endpoints included histomorphometric indices of bone formation and bone resorption (MS/BS, MAR, bone formation rate [BFR/BS], and remodeling‐, modeling‐, and overflow modeling‐based formation [RBF/BS, MBF/BS, oMBF/BS]) in the relevant bone envelopes (cancellous, endocortical, intracortical, periosteal) and were analyzed in the same manner as the analysis of the primary efficacy endpoint. The change and percent change in s‐PINP and s‐CTX from baseline were summarized descriptively. The relationship between bone turnover markers and histomorphometric parameters was analyzed using linear regression analyses. The analyses include scatter plots, Spearman's rank correlation coefficient (and 95% confidence interval [CI]), and slope of the regression line in a linear regression model. No formal statistical hypothesis testing was performed for safety endpoints and data are summarized descriptively. All descriptive and statistical analyses were performed using SAS statistical software version 9.4 (SAS Institute, Cary, NC, USA).

## Results

### Baseline demographics and characteristics

Twenty‐three subjects were enrolled in the study, all of whom were included in the safety population. Twenty subjects completed the study. Two discontinued because of AEs and 1 was discontinued because of incorrect dosing of fluorochrome labels. Biopsies were obtained from 20 subjects after 3 months of daily abaloparatide administration. Nineteen biopsies were evaluable for all indices except for 1 subject for whom cancellous bone was not evaluable and 1 subject for whom only cancellous bone was evaluable. Baseline characteristics were generally consistent with the patient population in the ACTIVE trial, although with moderately higher baseline lumbar spine BMD *T*‐scores (Table 1).

**Table 1:** Baseline Characteristics (Bone Biopsy Population)

Characteristic	Abaloparatide 80 μg (*N* = 19)
Age (years)	
Mean (SD)	67.4 (8.3)
Median (min, max)	65 (55, 85)
Age group, *n* (%)	
<65 years	7 (36.8)
65–<75 years	8 (42.1)
≥75 years	4 (21.1)
Race—white, *n* (%)	19 (100)
Weight (kg), mean (SD)	60.5 (8.2)
BMI (kg/m^2^), mean (SD)	24.1 (3.4)
BMD *T*‐score, mean (SD)	
Total hip	−2.33 (0.68)
Lumbar spine	−2.18 (1.25)
Femoral neck	−2.43 (0.57)
Prevalent vertebral fracture at baseline^a^	7 (36.8)
Prior clinical fracture^b^	7 (36.8)
25‐hydroxyvitamin D (nmol/L), mean (SD)	120.0 (31.0)

BMD = bone mineral density; BMI = body mass index; SD = standard deviation.

^a^ Prevalent vertebral fracture at baseline was reported based on review of spine radiographs.

^b^ Prior clinical fracture was reported based on medical history.

### Dynamic bone histomorphometry

**Fig 2:**
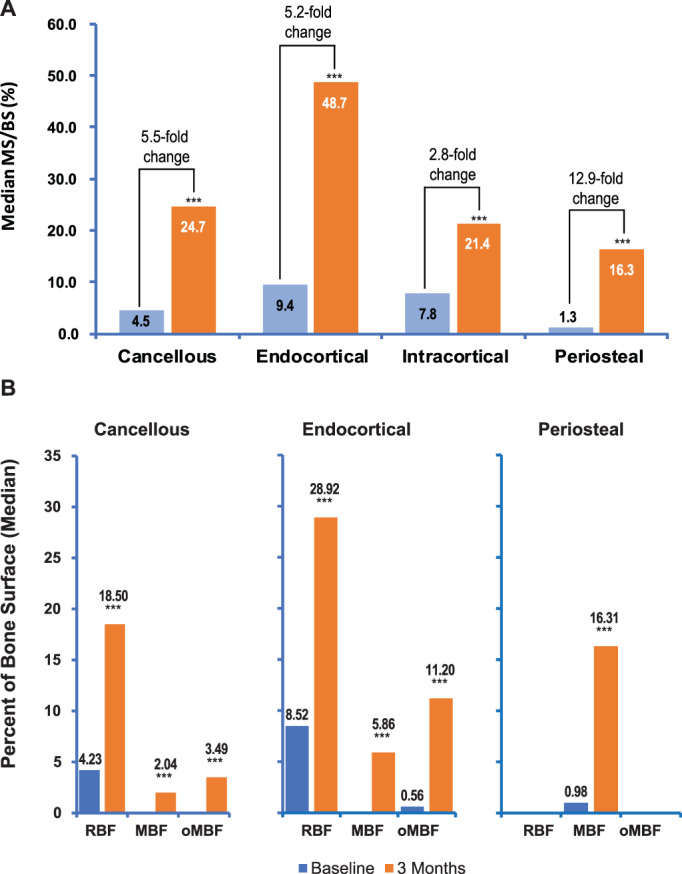
Bone formation at baseline and 3 months. (*A*) Median mineralizing surface per unit of bone surface at baseline and 3 months for the four bone envelopes. (*B*) Median bone formation at baseline and 3 months. Shown are remodeling‐based formation (RBF), modeling‐based formation (MBF), and overflow modeling‐based formation (oMBF) as a percentage of bone surface in the cancellous, endocortical, and periosteal envelopes. ***p < .001 within‐group changes from baseline to 3 months by paired *t* test (or Wilcoxon signed‐rank test instead, if the normality assumption of the data is not satisfied). MBF = modeling‐based formation; MS/BS = mineralizing surface/bone surface; oMBF = overflow modeling‐based formation; RBF = remodeling‐based formation.

At baseline, median MS/BS was less than 10% of total surface on all bone envelopes (4.5% for cancellous, 9.4% for endocortical, 7.8% for intracortical, and 1.3% for periosteal surfaces) (Fig. 2 *A*, Table 2). Median MS/BS increased to 24.7% for cancellous, 48.7% for endocortical, 21.4% for intracortical, and 16.3% for periosteal surfaces after 3 months of abaloparatide treatment, representing a 5.5‐fold increase in cancellous, 5.2‐fold increase in endocortical, 2.8‐fold increase in intracortical, and 12.9‐fold increase in periosteal surfaces (*p* < .001 versus baseline for all). MAR was significantly increased only on intracortical surfaces after 3 months of abaloparatide treatment, whereas BFR/BS was significantly increased on cancellous, endocortical, and intracortical surfaces (Table 2, *p* < .001 versus baseline for all). At the periosteal surface, 4 samples had double labels at baseline and 11 had double labels at month 3, thus MAR and BFR/BS are presented both with and without imputation. Imputed periosteal MAR and periosteal BFR/BS were significantly increased at month 3 versus baseline; nonimputed periosteal MAR and periosteal BFR/BS were not.

**Table 2:** Histomorphometric Dynamic Indices (Baseline to Month 3)

Indices	Cancellous		Endocortical		Intracortical		Periosteal	
	Baseline	Month 3	Baseline	Month 3	Baseline	Month 3	Baseline	Month 3
**MS/BS (%)**								
*N*	18	18	18	18	18	18	18	18
Median	4.51	24.66 ***	9.42	48.65 ***	7.77	21.40 ***	1.26	16.31 ***
(Q1, Q3)	(3.69, 6.83)	(13.70, 33.33)	(6.42, 18.06)	(40.33, 80.03)	(5.10, 12.08)	(14.02, 28.69)	(0.00, 2.19)	(10.22, 27.48)
**MAR (μm/d) imputed**								
*N*	18	18	17	18	18	18	12	18*
Median	0.512	0.500	0.520	0.549	0.665	0.841 **	0.300	0.335
(Q1, Q3)	(0.468, 0.592)	(0.469, 0.531)	(0.461, 0.595)	(0.502, 0.612)	(0.581, 0.696)	(0.712, 0.918)	(0.300, 0.343)	(0.300, 0.484)
**BFR/BS (mm** ^**3**^ **/mm** ^**2**^ **/yr) imputed**								
*N*	18	18	18	18	18	18	18	18
Median	0.009	0.043 ***	0.021	0.098 ***	0.019	0.066 ***	0.002	0.024 ***
(Q1, Q3)	(0.006, 0.015)	(0.023, 0.069)	(0.011, 0.039)	(0.078, 0.159)	(0.011, 0.033)	(0.041, 0.091)	(0.000, 0.003)	(0.012, 0.043)
**MAR (μm/d)**								
*N*	18	18	16	18	18	18	4	11
Median	0.512	0.500	0.527	0.549	0.665	0.841 **	0.369	0.482
(Q1, Q3)	(0.468, 0.592)	(0.469, 0.531)	(0.479, 0.596)	(0.502, 0.612)	(0.581, 0.696)	(0.712, 0.918)	(0.343, 0.639)	(0.352, 0.528)
**BFR/BS (mm** ^**3**^ **/mm** ^**2**^ **/yr)**								
*N*	18	18	17	18	18	18	10	11
Median	0.009	0.043 ***	0.023	0.098 ***	0.019	0.066 ***	0.000	0.043
(Q1, Q3)	(0.006, 0.015)	(0.023, 0.069)	(0.014, 0.039)	(0.078, 0.159)	(0.011, 0.033)	(0.041, 0.091)	(0.000, 0.002)	(0.022, 0.057)
**RBF/BS (%)**								
*N*	18	18	18	18	18	18	18	18
Median	4.23	18.50 ***	8.52	28.92 ***	7.77	21.38 ***	0.00	0.00
(Q1, Q3)	(3.57, 6.83)	(8.46, 22.09)	(5.63, 17.34)	(23.83, 38.57)	(5.10, 12.08)	(14.02, 30.10)	(0.00, 0.00)	(0.00, 0.00)
**MBF/BS (%)**								
*N*	18	18	18	18			18	18
Median	0.000	2.04 *** ^a^	0.000	5.86 *** ^a^			0.978	16.31 ***
(Q1, Q3)	(0.000, 0.120)	(0.928, 4.611)	(0.000, 0.518)	(3.015, 16.60)			(0.000, 2.193)	(10.22, 24.99)
**oMBF/BS (%)**								
*N*	18	18	18	18			18	18
Median	0.000	3.49 ***	0.560	11.20 ***			0.000	0.000
(Q1, Q3)	(0.000, 0.183)	(2.138, 4.552)	(0.000, 1.065)	(3.715, 21.08)			(0.000, 0.000)	(0.000, 0.000)

BFR/BS = bone formation rate; BS = unit of bone surface; MAR = mineral apposition rate; MS/BS = mineralizing surface; MBF/BS = modeling‐based formation; oMBF/BS = overflow modeling‐based formation; RBF/BS = remodeling‐based formation.

^a^ Did not pass normality assumption; thus, Wilcoxon signed‐rank test was used.

* *p* < .05; paired *t* tests (or Wilcoxon signed‐rank test instead, if the normality assumption of the data is not satisfied) were used to compare the differences between baseline and month 3.

** *p* < .01; paired *t* tests (or Wilcoxon signed‐rank test instead, if the normality assumption of the data is not satisfied) were used to compare the differences between baseline and month 3.

*** *p* < .001; paired *t* tests (or Wilcoxon signed‐rank test instead, if the normality assumption of the data is not satisfied) were used to compare the differences between baseline and month 3.

**Fig 3:**
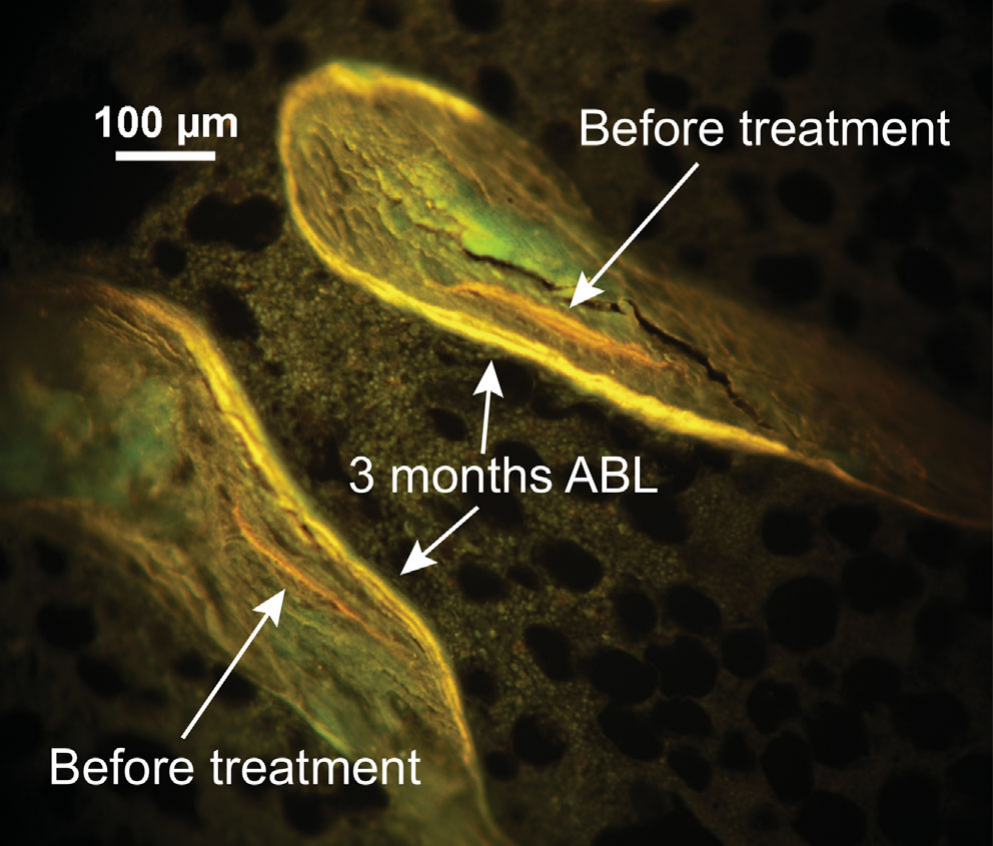
Increase in MS/BS and oMBF with abaloparatide treatment. ABL = abaloparatide; MS/BS = mineralizing surface/bone surface; oMBF = overflow modeling‐based formation.

**Fig 4:**
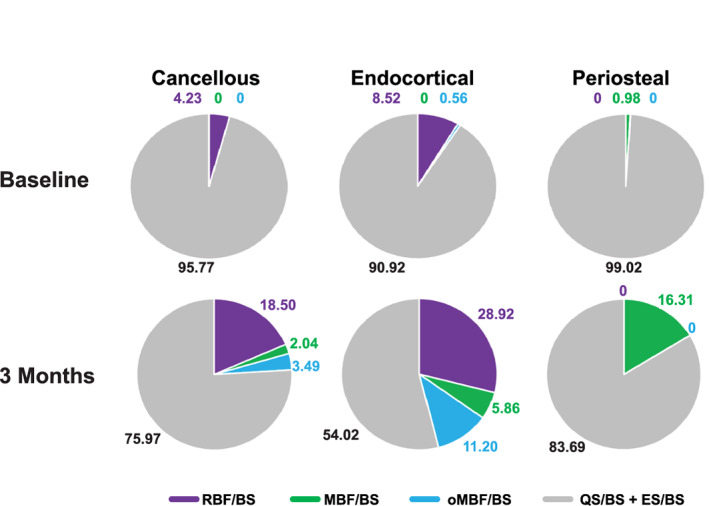
Percent bone formation at baseline and 3 months (median). Shown are remodeling‐based formation (RBF), modeling‐based formation (MBF), and overflow modeling‐based formation (oMBF) as a percentage of bone surface at 3 months by bone envelope. BS = bone surface; ES = eroded surface; MBF = modeling‐based formation; oMBF = overflow modeling‐based formation; QS = quiescent surface; RBF = remodeling‐based formation.

Fig. 3 illustrates the increases in mineralizing surface in cancellous bone with abaloparatide treatment, with greater month 3 tetracycline labeling present relative to the demeclocycline fluorochrome that was administered at baseline. At baseline, the group medians for MBF/BS and oMBF/BS were zero on all surfaces except for periosteal MBF/BS and endocortical oMBF/BS, which were less than 1% of total surface (Fig. 2 *B*, Table 2, Fig. 3). At month 3, significant increases were observed in MBF/BS on cancellous, endocortical, and periosteal surfaces (*p* < .001 versus baseline for all), with the greatest change observed for periosteal MBF/BS (0.98% to 16.31%) (Figs. 2*B* and 4). Significant increases were also observed for oMBF/BS versus baseline, to 3.49% and 11.20% of total surface on cancellous and endocortical surfaces respectively (*p* < .001 for both). RBF/BS was significantly increased from <10% at baseline to 18.50% on cancellous, 28.92% on endocortical, and 21.38% on intracortical surfaces at month 3 (*p* < .001 for all). Modeling‐based formation (MBF + oMBF) accounted for 37% and 23% of the increase from baseline in bone‐forming surface on the endocortical and cancellous surfaces, respectively.

### Correlations with serum biomarkers

**Fig 5:**
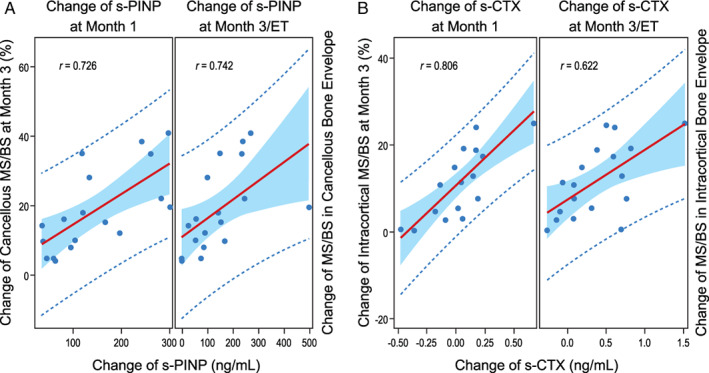
Correlations between changes in s‐PINP/s‐CTX and changes in cancellous/intracortical MS/BS. (*A*) Scatter plots of change in mineralizing surface per unit of bone surface (MS/BS) at 3 months by changes in s‐PINP at 1 month and 3 months. (*B*) Scatter plots of change in intracortical mineralizing surface (MS/BS) at 3 months by changes in s‐CTX at month 1 and month 3. ET = end of treatment; MS/BS = mineralizing surface/bone surface; s‐CTX = serum carboxy‐terminal cross‐linking telopeptide for type I collagen; s‐PINP = serum procollagen type I N‐terminal propeptide.

Changes from baseline (delta, Δ) in serum biomarkers at month 1 or month 3 were generally good surrogates for changes in bone histomorphometry endpoints from baseline to month 3 with abaloparatide (Fig. 5, Table 3). For change in MS/BS, strong positive correlations with month 1 or 3 Δs‐PINP were found on cancellous, endocortical, and intracortical surfaces (*r* = 0.62–0.82). Similar correlations were observed for Δs‐PINP versus ΔBFR/BS (*r* = 0.53–0.76) and for Δs‐CTX versus ΔMS/BS (*r* = 0.51–0.81) on these surfaces. No significant correlations were observed between serum biomarker and the more variable histomorphometric changes at the periosteal surface. For MBF and oMBF on cancellous and endocortical surfaces, positive correlations were also found between month 3 Δs‐PINP versus ΔMBF/BS (*r* = 0.49 and 0.59), oMBF/BS (*r* = 0.60 and 0.43), and RBF/BS (*r* = 0.75 and 0.73).

**Table 3:** Correlation Coefficients for Changes in Serum Biomarkers Versus Changes in Histomorphometric Indices of Bone Formation (Nonsignificant Values in Italics)

	Month 1	Month 3	Month 1	Month 3
	S‐PINP	S‐PINP	S‐CTX	S‐CTX
Cn.MS/BS	0.726	0.742	0.511	0.688
Ec.MS/BS	0.659	0.820	0.620	0.651
Ic.MS/BS	0.701	0.624	0.806	0.622
Ps.MS/BS	*0.270*	*0.449*	*0.125*	*0.494*
Cn.BFR/BS	0.748	0.736	ND	ND
Ec.BFR/BS	0.529	0.765	ND	ND
Ic.BFR/BS	0.664	0.701	0.740	0.616
Ps.BFR/BS	*0.314*	*0.143*	ND	ND
Cn.RBF/BS	0.738	0.750	0.517	0.723
Cn.MBF/BS	0.486	0.490	0.501	*0.377*
Cn.oMBF/BS	0.560	0.596	*0.389*	0.593
Ec.RBF/BS	0.583	0.730	*0.409*	0.567
Ec.MBF/BS	0.630	0.585	0.605	0.552
Ec.oMBF/BS	*0.147*	*0.427*	*0.344*	*0.328*

BFR/BS = bone formation rate; BS = unit of bone surface; Cn = cancellous; Ec = endocortical; Ic = intracortical; MBF/BS = modeling‐based formation; MS/BS = mineralizing surface; ND = not determined; oMBF/BS = overflow modeling‐based formation; Ps = periosteal; RBF/BS = remodeling‐based formation; s‐PINP = serum procollagen type I N‐terminal propeptide; s‐CTX = serum carboxy‐terminal cross‐linking telopeptide of type I collagen.

Correlations were generally similar for absolute values of serum biomarkers at months 1 and 3 versus month 3 MS/BS or BFR/BS on cancellous, endocortical, and intracortical surfaces, with correlation coefficients that tended to be lower. Eroded surface was positively correlated with s‐CTX at month 1 or 3 on the intracortical surface (*r* = 0.505 and 0.595, respectively) but not on cancellous or endocortical surfaces. No significant correlations were observed between cortical porosity and s‐CTX at either month 1 or 3.

### Safety

Eighteen subjects (78.3%) experienced at least one treatment‐emergent AE (TEAE). TEAEs that occurred in at least 2 subjects were dizziness (*n* = 4, 17.4%), nausea (*n* = 4, 17.4%), bursitis (*n* = 3, 13.0%), headache (*n* = 3, 13.0%), diarrhea (*n* = 2, 8.7%), upper respiratory infection (*n* = 2, 8.7%), fall (*n* = 2, 8.7%), wheezing (*n* = 2, 8.7%), and ecchymosis (*n* = 2, 8.7%). The majority of TEAEs were mild or moderate in severity; 1 subject experienced two TEAEs (nausea and vomiting) that were considered severe.

No deaths were reported during the study. Two SAEs (atrial fibrillation and vomiting) were reported in 1 subject each. Neither was considered to be treatment related; however, the vomiting event resulted in treatment discontinuation. One subject who experienced an AE of heart palpitations also discontinued treatment and withdrew from the study.

## Discussion

The current study demonstrates that treatment with abaloparatide for 3 months stimulates bone formation on cancellous as well as endocortical, intracortical, and periosteal surfaces in transiliac bone biopsies obtained from postmenopausal women with osteoporosis. These changes reflected increases in both remodeling‐ and modeling‐based bone formation, demonstrating for the first time that abaloparatide can stimulate bone formation directly on surfaces without prior resorption.

These results contrast with the findings from the pivotal ACTIVE trial where analyses of transiliac bone biopsies obtained after an average of 15.9 months of treatment with abaloparatide did not show significant increases in cancellous MS/BS or BFR/BS in either the abaloparatide or teriparatide groups versus placebo.^(^ ^10^ ^)^ Other reports have shown that these parameters were not increased with teriparatide treatment after 18 to 24 months,^(^ ^17^, ^19^ ^)^ in contrast to the significant increases observed in studies of shorter duration.^(^ ^7^, ^12^, ^20^ ^)^ The current study demonstrates time‐dependent effects with abaloparatide that are consistent with the serum biomarker profile that peaks within the first 3 months and slowly declines over the 18‐month treatment period.^(^ ^10^ ^)^ There are also prominent temporal changes in cancellous bone formation indices in bone biopsies from women receiving the sclerostin inhibitor romosozumab, with significant increases at month 2 followed by significant decreases versus placebo at month 12.^(^ ^11^ ^)^

A limitation of the prior bone histomorphometric assessment of the ACTIVE biopsies is that only a few cortical bone indices were assessed. The current study showed significant increases in bone formation on periosteal, endocortical, and intracortical surfaces after 3 months of abaloparatide treatment. This is consistent with nonclinical studies indicating increased periosteal and endocortical bone formation in mice and rats treated with abaloparatide for up to 12 months^(^ ^21^, ^22^, ^23^ ^)^ and increased endocortical bone formation in monkeys treated for 16 months.^(^ ^24^ ^)^ Clinical studies of teriparatide using the quadruple‐labeled technique also demonstrated increased periosteal and endocortical bone formation after 1 to 3 months of teriparatide.^(^ ^4^, ^7^, ^12^, ^20^ ^)^ In the AVA trial, 3 months of teriparatide increased median MS/BS by 6.4‐fold on the periosteal surface and 4.5‐fold on the endocortical surface relative to baseline,^(^ ^12^ ^)^ whereas in the current abaloparatide study, these medians were increased 12.9‐ and 5.2‐fold, respectively. In contrast, romosozumab did not significantly increase bone formation on the iliac periosteal surface after 2 months.^(^ ^11^ ^)^ The early increases in periosteal and endocortical MS/BS with abaloparatide are consistent with the rapid increases in BMD observed at the hip, increases that exceeded those of open‐label teriparatide at months 6, 12, and 18.^(^ ^9^ ^)^ The results from our study show that abaloparatide significantly increases mineralizing surfaces in all four bone envelopes. Therefore, in subjects with very low BMD, the response might be less if trabecular bone volume (BV/TV) is low and disrupted, but the cortices never disappear even in the most severe cases of osteoporosis, and greatest response was observed on the endocortical surface. The increase in BMD in response to treatment in subjects with very low BMD confirms the anabolic response to treatment. In ACTIVE, approximately 25% of women had a BMD *T*‐score of −3.5 or less at the spine or hip. Two reports confirm BMD increases in subjects with *T*‐scores of less than −3 and − 3.5, respectively.^(^ ^25^, ^26^ ^)^

Periosteal bone formation occurs predominantly via bone modeling, thus the robust increase in periosteal MS/BS with abaloparatide is itself an indication of stimulated bone modeling.^(^ ^3^ ^)^ Characterization of bone‐forming surfaces confirmed that the periosteal response to abaloparatide was modeling‐based, and modeling‐based bone formation also occurred on endocortical and cancellous surfaces. Both modeling and overflow remodeling increased on endocortical and cancellous surfaces with abaloparatide, with MBF representing direct activation of formation on quiescent surfaces and oMBF representing overflow of bone formation adjacent to an active RBF surface. These modeling increases with abaloparatide compared favorably with those of teriparatide in the AVA trial.^(^ ^4^ ^)^ Comparing group means as reported in AVA, MBF was increased from <0.6% at baseline to 10.9% with abaloparatide and 5.6% with teriparatide on the endocortical surface at month 3, whereas on the cancellous surface, month 3 MBF was 3.1% and 1.4%, respectively.

Romosozumab stimulated MBF on endocortical and cancellous (but not periosteal) surfaces in 2‐month biopsies,^(^ ^27^ ^)^ although a cross‐study comparison is challenging because of different methodologies, including a lack of separate oMBF analyses in the romosozumab study.

Abaloparatide treatment also increased RBF on cancellous and endocortical surfaces. Increased RBF may occur as a result of extending the duration of bone formation on active remodeling surfaces, an improvement in osteoblast efficiency, and/or an increase in forming surface secondary to increased remodeling rate. The positive correlation between s‐CTX and RBF suggests that increased remodeling rate likely contributed to the increase in RBF. Under the influence of abaloparatide, these RBF surfaces would be expected to fill to a greater extent than the depth of the erosion, thus providing a positive bone balance for each bone multicellular unit (BMU) that contributes to BMD gains. Although an imperfect surrogate of bone balance, wall thickness was numerically but not significantly higher in the abaloparatide group versus placebo in ACTIVE,^(^ ^10^ ^)^ and previous reports have demonstrated increased wall thickness with abaloparatide in animal models.^(^ ^24^ ^)^ In addition, the observed increase in remodeling rate would provide more opportunities for overflow MBF, which would further contribute to the increases in bone volume. Although the abaloparatide‐mediated increases in MBF and oMBF on the cancellous and endocortical surface were numerically smaller than for RBF, their impact on bone volume may be similar. Even if wall thickness is increased by 20% to 30%, as reported previously in animal models with abaloparatide^(^ ^24^ ^)^ or PTH,^(^ ^28^ ^)^ 70% to 80% of RBF would be acting just to refill resorption space. In contrast, all bone formed through MBF or oMBF represents net addition of bone volume. Thus, based on the effects of abaloparatide in the current study, the impact on cancellous bone volume of the increase from 0% at baseline to 5.53% at 3 months in modeling formation (MBF + oMBF) may actually exceed that of the increase from 4.23% at baseline to 18.50% at 3 months in remodeling formation (RBF).

Abaloparatide also increased bone formation on intracortical surfaces. The increase in intracortical MS/BS likely reflects an increase in intracortical resorption (and remodeling rate) with abaloparatide, consistent with the effects observed with teriparatide in the AVA trial.^(^ ^12^ ^)^ However, the increase in intracortical MAR suggests the rate at which these resorption spaces were being filled was more rapid, an effect that could minimize the potential increase in cortical porosity. Previous histomorphometry data obtained from mice and monkeys suggested that abaloparatide may not increase intracortical remodeling to the same degree as teriparatide.^(^ ^22^, ^24^ ^)^ Three‐dimensional modeling of hip dual‐energy X‐ray absorptiometry (DXA) scans from the ACTIVE trial also suggest that abaloparatide and teriparatide may differentially affect intracortical remodeling, as reflected in cortical volumetric BMD changes that were greater with abaloparatide.^(^ ^29^ ^)^ In the current study, the early increases in intracortical remodeling (MS/BS) in the iliac crest were positively correlated with increases in s‐CTX. Thus, regression of s‐CTX increases after month 3 would predict declines in intracortical remodeling with continued abaloparatide treatment, unlike the persistent CTX increases with teriparatide.^(^ ^12^ ^)^

Bone formation indices at cancellous and endocortical surfaces were positively correlated with serum biomarkers. Significant correlation coefficients were found between MS/BS, RBF, and MBF and s‐PINP or s‐CTX collected after 1 or 3 months of abaloparatide. These results suggest that serum biomarkers obtained as early as 1 month of treatment reflect a subject's response to anabolic therapy at the tissue level; such data may be useful to encourage compliance. Not all subjects will have baseline serum biomarkers measured before anabolic therapy, and for some subjects a recent fracture may complicate interpretation of their baseline values. Therefore, the observation that actual serum biomarker values reflect tissue level anabolism with abaloparatide may also be important. Early changes in serum biomarkers with abaloparatide have previously been shown to correlate well with BMD increases after 18 months of therapy.^(^ ^15^ ^)^ The current study thus connects the early tissue‐level increases in bone formation to later BMD increases through similar correlations to systemic biomarker responses.

There are several limitations within the current study. It was an open‐label, single‐arm study with no placebo group comparison and the study only included white participants. Data generated from bone biopsies are quite variable, not only between subjects but also between biopsies taken from the same subject.^(^ ^30^ ^)^ Thus, the quadruple labeling procedure provides internal control, allowing for a baseline comparison of bone formation indices within the same sample. This does limit the ability to evaluate the effect of treatment on bone microarchitectural endpoints, although such parameters would not be expected to change significantly after only 3 months of treatment. Cross‐study comparisons of the effects of different anabolic agents should be made with caution. However, the current study had a number of design features that were similar to the AVA trial, including the timing of the biopsies and the use of the same labeling protocol.^(^ ^12^ ^)^ The tissue processing and histomorphometry were also performed by the same individuals for both studies and the same clinical sites were used for both studies. Studies based on transiliac bone biopsies may not precisely reflect changes in bone at weight‐bearing sites. However, bone formation increases were demonstrated on cancellous and endocortical surfaces after 40 days of teriparatide in femur neck samples collected from subjects undergoing total hip replacement,^(^ ^31^ ^)^ suggesting that iliac bone biopsy data can reflect similar anabolic effects throughout the skeleton.

In conclusion, 3 months of daily administration of abaloparatide resulted in increased bone formation across all four bone envelopes in the iliac crest. These increases were associated with stimulation of both modeling‐based and remodeling‐based bone formation. The changes in bone formation parameters are consistent with the serum biomarker response to abaloparatide, which is associated with robust increases in bone mass in the hip and spine. The consistent and rapid anabolic effects at the tissue level provide important new insights into the mechanism of action of abaloparatide in women with postmenopausal osteoporosis and likely contribute to its demonstrated antifracture effects.

## Disclosures

DWD has received research support from Amgen, Eli Lilly, and Radius Health, Inc. He has received consulting and speaking fees from Amgen, Eli Lilly, and Radius Health, Inc. HZ has received research support for the study from Radius Health, Inc. SDR has received research support for the study from Radius Health, Inc. CR has received research support and grants from Amgen, CytoDyn, Eli Lilly, Genentech, Integra, Novartis, Radius Health Inc., Roche, and Senhwa. PDM has received research support for the study from Radius Health, Inc. MSO was an employee of and held equity in Radius Health, Inc. MA and BHM are employees of and hold equity in Radius Health, Inc.

### Peer Review

The peer review history for this article is available at https://publons.com/publon/10.1002/jbmr.4243.
